# Acute mountain sickness amongst tourists to Lhasa

**DOI:** 10.1186/s13690-016-0172-6

**Published:** 2017-01-27

**Authors:** Gaurav Sikri, Srinivasa Bhattachar

**Affiliations:** Department of Physiology, Armed Forces Medical College, Wanowarie, Sholapur Road, Pune, Maharashtra 411040 India

## Abstract

Acute mountain sickness is the commonest acute high altitude illness occurring at high altitude. Its prevalence is dependent on the ascent rate, altitude achieved, physical effort required to reach the target altitude and pharmacological intervention undertaken by the tourists visiting high altitude areas. This Letter to the Editor is an endeavour to re-emphasise the importance of all these factors affecting the prevalence of acute mountain sickness.

To the Editor

We read with profound interest the article titled ‘Acute mountain sickness among tourists visiting the high-altitude city of Lhasa at 3658 m above sea level: a cross-sectional study’ by Gonggalanzi et al. [1]. Indeed this study is one of its kind, which involved evaluation of a large sample size of ordinary travellers to Lhasa for acute mountain sickness (AMS). As reported by the authors, 47.3% (1022 out of 2160) tourists travelled to Lhasa by means other than air (by road, rail and train) and 35.9% of them suffered from AMS. It would be interesting to know the ascent profile and travel history of these subjects. Their travel details like starting altitudes, travel time and average ascent rates would have elucidated further the effects of mode of travel/induction to high altitude (HA) on occurrence of AMS as many of the travel related symptoms in HA terrain like fatigue, motion sickness etc. may imitate symptoms included in questionnaire based on Lake Louise scoring system (LLSS).

Authors have mentioned that 46.4% (965 out of 2081) of the tourists used various prophylactic agents in the present study. As per Wilderness Medical Society guidelines, the prophylactic agents generally used against prevention of acute high altitude illnesses are classified into two groups: one group consisting of drugs used against AMS/high altitude cerebral edema (acetazolamide, steroids) and other group having drugs used against high altitude pulmonary edema (nifedipine) [2]. Evaluation of effects of these drugs as one combined group by the authors and reporting of occurrence of AMS does not bring out their actual relationship. It would have been nice if authors had evaluated the relationship between AMS and drugs used specifically against it. Also, elaboration on history of drug intake like timings of starting/stopping medication and dosage would have explained this relationship better in travellers to Lhasa.

As acknowledged by the authors, AMS is generally known to occur 6 to 12 h after ascending beyond 2500 m [3]. But the data presented in this study may not actually support this because of a possible ‘recall’ bias because of lack of uniformity in filling and submitting questionnaires based on LLSS. It would have been interesting if the tourists were asked to fill the questionnaires after a specific time say after 12 or 24 h of ascent to 3658 m. This could have reduced this possible bias and helped in comparison of outcome of the present study with other studies at similar altitudes.

## Abbreviations

AMS: Acute mountain sickness
HA: High altitude
LLSS: Lake Louise scoring system
